# Supplementary material for: Investigating the association between body fat and depression via Mendelian randomization
Source: Transl Psychiatry. 2019 Aug 5;9:184. doi: 10.1038/s41398-019-0516-4 (PMC6683191; doi:10.1038/s41398-019-0516-4)
Supplement: Supplementary file 1 — Supplementary Figure 1 [file 41398_2019_516_MOESM1_ESM.docx]

Supplemental Material

**Supplementary Figure 1: Genetic correlations.** Genetic correlations between the 21 anthropometric from the UK Biobank.
